# Priority setting and cross-country learning: the relevance of TO-REACH for primary care

**DOI:** 10.1017/S1463423622000287

**Published:** 2022-07-22

**Authors:** Peter Groenewegen, Johan Hansen, Nick Fahy, Alexander Haarmann, Sabrina Montante, Natasha Azzopardi Muscat, Mircha Poldrugovac, Walter Ricciardi, Gianpaolo Tomaselli

**Affiliations:** 1 Netherlands Institute for Health Services Research (NIVEL) and Utrecht University, Departments of Sociology and Human Geography, Utrecht, Netherlands; 2 Netherlands Institute for Health Services Research (NIVEL), Utrecht, Netherlands; 3 European Observatory on Health Systems and Policies, Brussels, Belgium; 4 Netherlands Institute for Health Services Research (NIVEL), Currently at Hertie School – Berlin’s University of Governance, Berlin, Germany; 5 Instituto Superiore di Sanità, Rome, Italy; 6 Department of Health Services Management, University of Malta, Msida, Malta; 7 National Institute of Public Health of Slovenia, Currently at Amsterdam UMC, University of Amsterdam, Amsterdam, Netherlands; 8 Catholic University of the Sacred Heart, Rome, Italy; 9 University of Malta, Msida, Malta

**Keywords:** international comparison, primary care, priority setting, transferability

## Abstract

**Aim:**

To inform the primary care community about priorities for research in primary care as came up from the European project TO-REACH and to discuss transferability of service and policy innovations between countries.

**Background:**

TO-REACH stands for **T**ransfer of **O**rganizational innovations for **R**esilient, **E**ffective, equitable, **A**ccessible, sustainable and **C**omprehensive **H**ealth services and systems. This EU-funded project has put health systems and services research higher on the European agenda and has led to the current development of a European ‘Partnership Transforming Health and Care Systems’.

**Methods:**

To identify research priorities, both qualitative and quantitative approaches were used. Policy documents and strategic roadmaps were searched, and priorities were mapped. Stakeholders were involved through national roundtable consultations and online consultations. Regarding transferability, we carried out a review of the literature, guided by a conceptual framework, and using a snowballing approach.

**Findings:**

Primary care emerged as an important priority from the inventory, as are areas that are conducive to strengthening primary care, such as workforce policies. The large variation in service organisation and policy around primary care in Europe is a huge potential for cross-country learning. However, the simple transfer of primary care service and policy arrangements from one health system to another has a big chance to fail, unless known conditions for successful transfer are taken into account and gaps in our knowledge about transfer are resolved.

## Introduction

Health systems share challenges, such as increasing and changing health care needs in an ageing population, workforce shortages, and cost increases. Various solutions have been proposed and implemented, and health systems can learn from each other in this respect. In an European Union (EU)-funded support action, called TO-REACH, priority areas where changes are needed have been addressed, as well as the question how health systems can effectively learn from each other in transferring solutions from one health system to another. In this paper, we present and discuss the results of this support action, focussing on primary care.

TO-REACH stands for **T**ransfer of **O**rganizational innovations for **R**esilient, **E**ffective, equitable, **A**ccessible, sustainable and **C**omprehensive **H**ealth services and systems. It is an EU-funded project that aimed to put health systems and services research (HSSR) higher on the European agenda (Walshe *et al.*, 2013). It had 28 partners from 20 countries, including EU member states, other European countries, and countries outside Europe. The project has contributed to putting HSSR on the European research agenda in the new framework programme Horizon Europe and led to the development of the Partnership Transforming Health and Care Systems. This future partnership is highly relevant for primary care. It will be a platform for developing and funding cross-national studies to improve health care policies and services, including primary care.

The TO-REACH project has two important outputs that can be used to inform the future research agenda for HSSR and that we will apply to primary care. They are published as Policy Briefs and based on more extensive research reports. The two policy briefs form the basis for this article. The first relates to the priorities for research. This part of the project output addresses which areas of policy and service innovation should be priorities (Hansen *et al.*, 2021). Among the broader priority areas that emerged from the project, primary care played an important role, as this is highly interconnected with other sectors, the first point of contact for citizens, and one of the most effective means of providing health care. The second output relates to cross-country learning and the transfer of innovations in policy and service delivery from one country/health system to another. This part focuses on what we know and on the gaps in our knowledge (Nolte and Groenewegen, 2021). We will elaborate these two lines in this paper by answering the following questions:

- What are priorities for HSSR, in general and from a primary care perspective?

- What do we know about and what are gaps in our knowledge of transferring policy and service innovations in primary care from one country to another?

The first question has been answered by analysing documents and consulting stakeholders about their priorities; the second question has been approached through a scoping review of the literature.

## Methods

The results presented in this paper have been reached in a systematic way with a different approach for priority setting and for cross-country learning and transfer. We have brought in the primary care perspective through additional literature and the experience of the authors.

### Priority setting

In accordance with traditional priority-setting approaches (Lomas *et al.*, 2003, Viergever *et al.*, 2010), both qualitative and quantitative methods were used to identify research priorities. Policy documents and strategic roadmaps at national and international levels, published between 2012 and 2018, were searched (Hansen *et al.*, 2019, Annex 5 Search Terms for Mapping of Reviews and Research Agendas). Also, roadmaps and conclusions from major international and EU-funded projects were analysed, and priorities were mapped.

Stakeholders, including patient organisations, were involved through national roundtable expert consultations in TO-REACH partner countries, with 15 consultations covering 14 of the project’s partner countries. Moreover, an online consultation was held among the wider scientific and stakeholder communities, with over 600 responses from 40 countries. The online consultation contained closed questions with predefined answering categories and questions inviting open answers. Participation invitations were disseminated by numerous means, including through newsletters and social media accounts of partner (eg, European Public Health Association (EUPHA); European Health Management Association (EHMA)); and non-partner organisations (eg, non-governmental organisations (NGOs) and the European Commission). Responses were primarily from European countries, but also included the USA, Canada, and Israel.

### Transfer of innovations

We carried out a review of the literature, guided by a conceptual framework (see results section below) and assessed the relevant literature in the context of this framework. This approach was informed by our knowledge of the available evidence in the field of policy transfer. This field of enquiry tends to lack empirical studies using rigorous, comparable designs, therefore precluding a more traditional systematic review of the evidence that seeks to combine studies in a meta-analytical framework. There is, however, a considerable evidence base in the related field of implementation and diffusion of innovation research that has brought together the theoretical and empirical evidence (Greenhalgh *et al.*, 2004). This provides a foundation for our review, which seeks to synthesise the evidence derived from conceptual studies of policy transfer and lesson-drawing with existing reviews and other studies on elements of the transfer process in an integrative (Whittemore and Knafl, 2005) or conceptual approach (Nutley *et al.*, 2002). This enabled us to identify the main knowledge gaps in the field of transfer of service and policy innovations.

We used, mainly, a snowballing approach, starting from seminal papers known by the authors or recommended by members of the project. We reviewed literature in the field of health services and systems research specifically, while also considering the wider social and political sciences literature and evaluation studies. The focus of the review was on transfer between health systems, although some of the literature derives from and/or is also relevant to transfer between health care organisations or regions.

## Results

### Priorities for research

**Figure 1.**
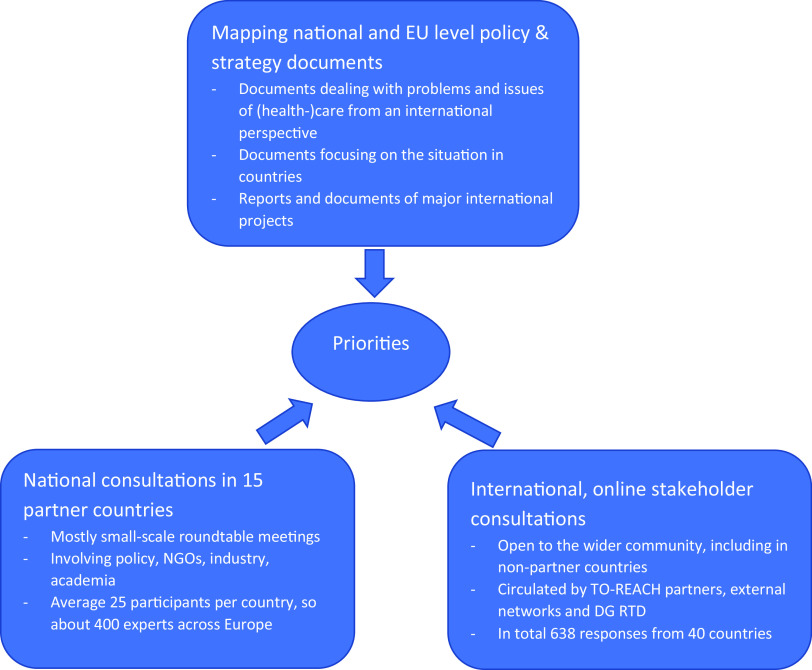
Inputs for priority setting

**Figure 2.**
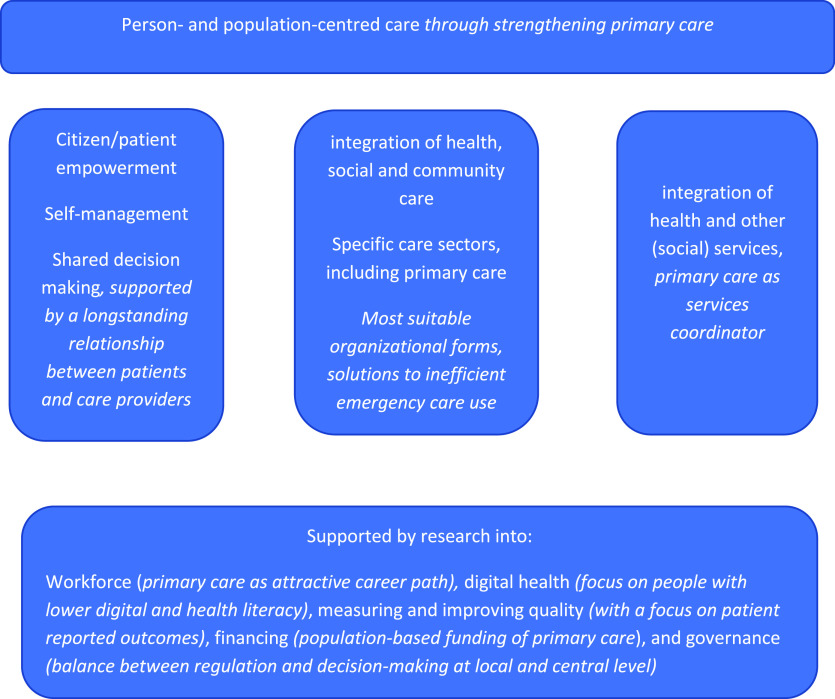
Key topics for learning across health systems with issues specifically important for primary care in *italic*

The inventory of research priorities aimed to get a broad input from different sources and stakeholders (see Figure 1). The resulting priorities were clustered in four overarching groups. Further research into person and population centredness of care has emerged as the main priority, followed by integration of health and other (social) services as a facilitator to achieve it. A third priority relates to specific health care sectors where important gaps in knowledge exist: long-term care, hospital care, primary care, and mental health care. The final set of priorities concerns research into supporting mechanisms across all sectors: workforce, digital health, measuring and improving quality, financing, and governance (see Figure 2). The question is how these may facilitate improvements in person- and population-centred care, care integration, and specific sectors, such as primary care. Below we address each of these four areas and describe research priorities for primary care.

### Improving person- and population-centred care

This is an important priority, and a way to achieve it is by strengthening primary care. The World Health Organization (WHO, 2015) describes it as ‘putting the comprehensive needs of people and communities, not only diseases, at the centre of health systems, and empowering people to have a more active role in their own health’. This is part of the paradigm shift from treating diseases to supporting patients and to person- and population-centred care. Person- and population-centred care requires a stronger orientation towards the needs of people in health care planning, organisation, and services. To do so, patients (and citizens) have to be empowered and primary care can play a role in this by supporting self-management, by involving people in decisions about their health and care, working towards co-production, by taking people’s health capacities into account and improving their health literacy, and by supporting informal carers. In particular, primary care systems where a longstanding relationship between patients and primary care providers is institutionalised, for example by a list system and gate-keeping, are able to support this. Health systems differ in how far they are in this respect, providing opportunities for cross-country learning. Primary care can be leading this paradigm shift. This asks for knowledge about the transfer of innovations between sectors and health systems (see next section).

Population orientation implies that primary care has to cross traditional boundaries (for example with public health) by focusing more on health promotion and disease prevention. This also requires an outreaching approach to actively seek out vulnerable groups, such as frail elderly who increasingly stay put in their own home.

### Integration of health and other services

The priority setting exercise showed that integration of services requires further research. A fragmented service, lacking in coordination and information sharing, can quickly reduce care quality and cost efficiency, and can harm patients. Integrated services are harnessed as a solution, easing access and increasing overall care quality. Integration is a key requisite for person-centred care, as illustrated by the WHO Framework on Integrated Health Services Delivery (WHO, 2016). Research into the integration across traditional health sectors is necessary because of changes in demography and epidemiology. Increasing life expectancy and the rise in multi-morbidities require health services, social care, and informal care to collaborate to improve life quality for those affected. In particular, there is a need for research to understand the link between the conditions necessary for local implementation and supportive macro policies. Integration cannot reach its full potential when confined to the health care sector. Health problems caused by housing, family relations, schooling, etc., can only be tackled alongside non-health care sector services. Consequently, research into the integration of health, social, and community care is required.

### Sectors of health care

Among the sectors of health care that were frequently mentioned as priority areas for research, primary care stands out. Modernising primary care systems was often presented as a solution for health system challenges related to ageing, multimorbidity, and cost containment. Combining the previous two broad areas, primary care can incorporate a person- and population-centred focus, comparatively easily, including aspects of health promotion and disease prevention, making it suited to assuming the role of services coordinator. However, these advantages must be developed through focused policies, based on research.

Strengthening primary care is a priority in countries with weak primary care and a traditionally strong hospital sector, with often a high utilisation rate of specialist care. However, the ongoing development of primary care is also a priority in countries with a strong primary care system. Both groups of countries are seeking solutions to similar problems, such as the most suitable form of primary care in a given community context (eg, depending on population density) or what tasks should be incorporated in primary care, particularly regarding hospital and specialist services. Another problem is ineffective emergency care use, due to difficulties in access to primary care outside office hours services or to financial reasons. This is an example where comparative research and learning across European health systems allows the implementation and evaluation of improved organisational models.

Primary care-specific gaps in knowledge are closely related to the mechanisms that support primary care in improving its role in person- and population-centred care and integration of health and other services. A notable example of such a primary care supportive mechanism is workforce development, including optimal team composition. Increased demands on primary care elicit discussion about the skill-mix in primary care teams and the required competences of primary care professionals, including inter-professional cooperation competences. The range of services provided in primary care differs considerably between countries, as does the availability of infrastructure, equipment, and ICT support. In countries with less strong primary care (Kringos *et al.*, 2013), the issue is how to steer the flow of patients away from specialist, emergency, or hospital care in a way that will be acceptable to patients. Research into adequate incentives for patients and care providers is important here. One commonality across countries is the primary care workforce shortage. How to make primary care a more attractive career path for young professionals is an important area of research and policy.

Accessibility of care is of prime importance, and people should have quick access. Given the large variations in waiting times for appointments with care providers in Europe, also for primary care, addressing long waiting times is a priority. The causes of waiting times in primary care are diverse, and hence, solutions are context-dependent. However, one solution is to make services more efficient to meet the increasing health needs of the population. Lack of primary care personnel may be responsible for long waiting times, in particular in deprived areas in cities and in remote rural areas. This requires extra investments in these areas and/or a new division of tasks between primary care professionals. Long waiting times may lead to additional problems, including corruption to circumvent waiting lists.

The national and online consultations identified several problems and questions around the organisation of the primary care sector, for example into the balance between specialism and generalism. Many roundtable discussions also identified macro-level governance and financial barriers; for example, resources remain too focused on secondary rather than primary or preventative care, and primary care payment mechanisms may be a barrier.

### Supporting conditions

Research into the supporting conditions for the priority areas mentioned above has been mentioned frequently. Five areas stand out:

- Adequate workforce, skill-mix, tasks, and responsibilities have already been mentioned in relation to primary care. The variation in primary care between health systems provides the opportunities for cross-national comparison and evaluation.

- Adequate ICT for health. One basic area is the support of administrative tasks and processes. The widespread introduction of electronic patient records helps exchange information between different providers, can support decision-making by health professionals, and thereby improve health care quality. A second area is ICT communication. Telemedicine is crucial in remote areas, both between professionals and patients and between professionals of different specialisation. The COVID-19 pandemic has shown that this is not restricted to remote areas and an important question is what will be maintained after the pandemic has resided and how unintended consequences will be addressed. A third area for primary care is the use of ICT to support patients in self-management and to empower patients through giving them access to their own records. However, E-health cannot fully replace personal contact with health professionals, especially for those with lower digital or health literacy. Finally, ICT use generates data that can be used in research and evaluation. However, the analysis of big data still requires the solution of major issues around standardisation, interoperability, and data exchange.

- Quality and suitable ways to measure it. Important from the perspective of primary care is a shift from the focus on quality of services to quality of life. This can be seen as a consequence from the paradigm shift to person- and population-centred care and raises questions about the responsibilities of health care and the integration of health care and social care. It also implies moving towards factors that matter for people (as reflected in the growing importance of patient-reported outcome measures (PROMs) and patient-reported experience measures (PREMs)). In particular, PROMs require further development to be useful for day-to-day primary care (OECD, 2019).

- Achieving better financing. With growing policy interest in value-based care models and outcome-based payments, population-based funding for health care in general and for primary care in particular is being discussed. Improved funding also implies prioritising sustainable, affordable funding of health services, and universal coverage.

- Employing good governance. Governance is only occasionally discussed as a priority. However, it is of particular importance in the context of integrating services and collaboration over the traditional boundaries of health systems. The balance between regulation and decision-making at local and central level poses questions where HSSR may be helpful. This is particularly relevant for primary care that has to take into account the local situation within a broader regulatory context (Groenewegen *et al.*, 2002).

### Transfer

Health systems in Europe face numerous challenges, many of which are the same in different countries, and there is an urgent need for innovative solutions to ensure that they continue to provide accessible health and long-term care that is of high quality, responsive, affordable, and financially sustainable. As discussed in the previous section, primary care is an area of high priority, and the large variation in ways of organising primary care shows the potential for health systems to learn from each other. European health care systems are a natural laboratory for HSSR (Groenewegen, 2013).

**Figure 3.**
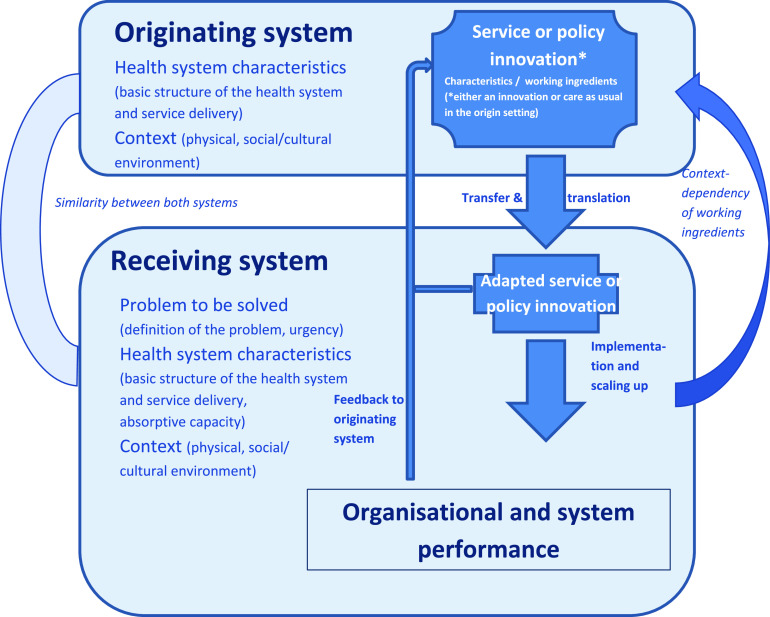
Conceptual framework of transfer of innovations between health systems

Our review of the literature was guided by a framework, based on work by Nilsen (Nilsen, 2015) that analysed existing reviews of implementation, including the comprehensive review by Greenhalgh et al (Greenhalgh *et al.*, 2004) and building on the seminal work by Rogers (Rogers and Shoemaker, 1977) on the diffusion of innovation. These distinguish the characteristics of the innovation, the characteristics of the users of the innovation, be they persons or organisations, and the process of adoption and implementation. Our focus is on the transfer between systems and not on the diffusion process as such. What is transferred are *health service and policy innovations*. Innovation is most often linked to technologies, but there are also non-technological innovations, including organisational innovations. Organisational innovation refers – for short – to new ways of organising practices, and new policies aimed to implement or regulate innovations (a discussion of the multidimensional concept of innovation is in section 2 of the policy brief (Hansen *et al.*, 2021) and in the report of the Expert Panel on Effective Ways of Investing in Health (EXPH, 2016)). Figure 3 provides this framework for understanding transfer of health service and policy innovations.

We distinguish between an originating system, where the innovation was invented and/or is already practised, and a receiving system, which intends to adopt the innovation. What is seen as an innovation in the receiving system may be actual practice in the originating system. An example is a patient list for GPs which is longstanding practice in a number of health care systems, such as the UK and the Netherlands, but which would be an innovation in others, such as Austria. It is important to be aware of the health system characteristics and the context in the originating and receiving systems. These may affect the need for adaptations of the innovation and the success of the transfer. The framework thus moves beyond simplistic models of ‘learning from best practice’, which often implied that what works in one context can simply be copied to another. In contrast, a model of ‘good practices’ highlights a relative fit to a particular context as well as the possibility of mutual learning. In that line, the experiences in the receiving system may be valuable for the originating system, creating the possibility of a feedback loop. The figure also emphasises the importance of evaluating the impact of an innovation on system performance. Again using the example of gate-keeping, introducing it in a health care system where GPs do not have the competences and support to treat health problems themselves would not effect in better performance of the system, but only in referrals. Finally, the problem that is to be solved by a health service or policy innovation should be thoroughly analysed. The ‘diagnosis’ of the problem is of paramount importance to be able to assess whether a problem is sought for a solution or a solution for a problem.

### Current knowledge on service and policy transfer

The review of the literature shows that it is easier to adopt and implement innovations that have a clear-cut advantage in (cost) effectiveness. When the problem that has to be solved is clear and the innovation addresses this and when the sociocultural context in which the innovation will be implemented is well-understood, the chances are bigger that transfer will be successful. Primary care out-of-hours care through (large) cooperatives is an example of an organisational model that quickly became the dominant model in Europe, partly through upscaling of an existing model, namely rota groups (Steeman *et al.*, 2020). Simple transfer is usually not successful; innovations have to be translated and customised to improve the ‘fit’ with local conditions. This requires a good understanding of the innovation itself; of how the innovation interacts with its context; and of the process of transfer itself. Experts and decision-makers, individuals, organisations, and networks, all play a role in innovation transfer and diffusion. Securing their commitment encourages success. As an example, medication review for elderly patients with complex care needs in primary care was introduced in Slovenia with support of the national health insurance fund. The team-based model that is used in the Netherlands was chosen for piloting and later implementation in Slovenia (Stuhec, 2021).

### Gaps in knowledge

It is unclear which particular health system characteristics and wider context elements are conducive to adopting, implementing and sustaining service and policy innovations. We know that context matters, but lack an understanding about what elements exactly matter (Schloemer and Schröder-Bäck, 2018). The structure of health systems may be important, but also aspects of the social and cultural context may impact on successful transfer. Primary care, with its central role in person and population centredness and in integration beyond the health system, is particularly sensitive to the social and cultural context. Cultural differences play a role in the transfer of innovations, but there is lack of knowledge of the mechanisms behind their impact and how to conceptualise and measure them. For example, extended nursing roles in primary care are likely to be affected by cultural attitudes of GPs, nurses, and patients (Kuhlmann, 2006). There is a need for better evidence on organisational arrangements to support service innovation at different levels and their impact on the transfer of service and policy innovations. Before innovations are transferred, there should be evidence of their effectiveness; however, the nature and level of the evidence needed are unclear, partly due to the relative novelty of some of the innovations to be transferred. This lack of evidence includes knowledge about the impacts of service and policy innovations on health system performance, including any unintended consequences. There is, for example, a big need for evidence-based interventions to attract primary care personnel to remote and rural/under-served areas. Overall, the evidence base on interventions to correct imbalances in primary care in rural areas is narrow. Findings are limited by a general lack of sufficient methodologically sound research (Roberts, 2017). Furthermore, the available evidence is biased towards programmes targeting physicians. This is even the case in task shifting programmes, where the availability of nurses might be the next problem to be solved, when these programmes were introduced without taking the availability of nurses into account (Bosmans *et al.*, 2021).

Finally, much work has to be done to further develop the research methodologies that can best advance cross-country learning, including how to identify countries for comparison; how to handle context; and addressing measurement problems. An approach to group countries/health systems into groups that could learn from each other was provided in the context of workforce policies (Batenburg, 2015).

Collaborative European research has to focus on aspects of innovation transfer that need to be understood better. This would provide a solid basis for addressing the challenges of system transformation and would help to maximise learning between European health systems to the benefit of primary care. In turn, results would not only ease the process of adopting innovations for receiving countries or rather deciding against a transfer. It could also open up debate about the claim that countries are ‘too special’ to transfer innovations to and that there is not sufficient evidence for the benefits of strong primary care.

## Discussion

From the inventory of the TO-REACH project, primary care emerges as an important priority, as are areas that are conducive to strengthening primary care, such as workforce policies. The large variation in service organisation and policy around primary care in Europe is a huge potential for cross-country learning. The service and policy challenges facing health systems across Europe have much in common; however, there are also differences between settings within and across countries which require suitable research designs, including targeted comparisons between a few countries. The priority areas that resulted from TO-REACH are consistent over time (Hummers-Pradier *et al.*, 2009, Schäfer *et al.*, 2011). This is not to say that nothing has changed. Rather, health systems are struggling to address the challenges in constantly changing contexts, including the current COVID-19 pandemic. Therefore, priority setting is not a one-off activity but requires continuous refinement to translate them to policy-relevant research questions.

Lessons from TO-REACH show that the simple transfer of primary care service and policy arrangements from one health system to another has a big chance to fail, unless conditions for successful transfer are taken into account and knowledge gaps about transfer are resolved. Also, the decision not to adopt a service or policy innovation from another health care system is important if it can be taken on the basis of evidence and experience; learning also means deciding not to transfer a service or policy innovation.

We still lack robust evidence on the particular characteristics of health systems that encourage innovation transfer and on the absorptive capacity of health systems to adapt and adopt service or policy innovations. Specific for the absorptive capacity at organisational level within primary care is that the size of primary care organisations can range considerably (from single-handed GP practices in some countries to large multi-disciplinary organisations in others). As is the case with other care sectors, primary care is strongly dependent on the surrounding health care network and the relations between different providers. Moreover, primary care is influenced by the incentives embedded in payment and funding systems that enable (or hinder) the successful implementation of evidence-based innovations.

The COVID-19 pandemic has reinforced the need for European health systems to learn from each other. It has shown the importance of primary care and indicated a number of areas where innovation and improvement are possible. First and foremost, the role of primary care in the response to the COVID-19 pandemic differs greatly between European countries and therefore provides opportunities for cross-country learning (EXPH, 2020). The pandemic has worked as a catalyst to become aware of existing problems and has thereby shown the importance of transformation of health care systems, for example further integration of parts of the health care system with social care (Kinder *et al.*, 2021). Care processes have changed through the quick introduction of innovations in the use of ICT, particularly electronic consultations. Pandemic response has also been negatively affected by lack of integration of and coordination between parts of the health care systems, in particular public health and primary care. The speed with which some lessons have been shared and implemented during the pandemic highlights how slow existing processes normally are. However, the challenges of learning from each other have also been highlighted, with a lack of clear means to identify the best innovations, how they exist within their organisational and system contexts, what is needed to transfer them elsewhere while preventing unintended consequences, and an overall lack of capacity for carrying out these tasks. While the TO-REACH project work was carried out before the pandemic struck, the challenges and potential solutions this project has identified will be even more relevant in the future reshaped by COVID-19.

The TO-REACH project has put HSSR on the European research agenda in the new EU framework programme Horizon Europe. The second pillar of the programme addresses global challenges. One of these is health, and under this heading, health systems are explicitly mentioned. This is the first time health systems is a separate research area in the framework programmes. Also, transnational cooperation and a new approach to international partnerships are addressed. The first calls under the new Horizon Europe framework programme have been published (https://ec.europa.eu/info/funding-tenders/opportunities/docs/2021-2027/horizon/wp-call/2021-2022/wp-4-health_horizon-2021-2022_en.pdf), with a part on ‘Ensuring access to innovative, sustainable, and high-quality health care’. As the work programme states: ‘Under this destination, research and innovation aims at supporting health care systems in their transformation to ensure fair access to sustainable health care services of high quality for all citizens’. This provides room for research into primary care. Also, the EU4Health Programme (DG Santé), inspired by the COVID-19 pandemic, stresses the need to strengthen the resilience and sustainability of health systems, to protect people in the EU from serious cross-border threats to health and to upskill health care and public health workforce (https://ec.europa.eu/health/sites/default/files/funding/docs/eu4health_factsheet_en.pdf).

The most important development, coming out of the TO-REACH project, is the current development of a ‘Partnership Transforming Health and Care Systems’. This will be a vehicle for research funding by the participating countries. There will be great opportunities for international comparative studies in primary care organisation, service delivery, and policy, based on the needs of the participating countries and funded by them with EU co-funding. These developments are supported by the fact that primary care has just been endorsed as the key priority programme within the WHO EURO programme of work 2022–2023 (WHO, 2012). The role of WHO is important to the EU’s work with countries in the EU neighbourhood that need to strengthen their primary care systems.

### Limitations

We have to consider some limitations of the analysis presented here. The TO-REACH project was not designed specifically for primary care. If this had been the case, priorities might have differed from the more general ones that our inventory showed. In the end, the relevance for primary care is our own interpretation of the results.

The focus of the TO-REACH project was on European countries and some high-income non-European countries. Priorities in low- and middle-income countries may differ from the ones described here. The general principles of transferability will largely the same; however, transferring service and policy transformations between countries with different levels of resources (eg, from high to low income countries) may require more adaptations or prove impossible.

Another limitation is that the inventory of priorities and the scoping review about transferability were made before the COVID-19 pandemic. As argued here, the pandemic may have affected priorities and ideas about cross-country learning.

## Conclusion

The basis for the proposed ‘Partnership Transforming Health and Care Systems’ is a willingness to share experiences and improve health care. This is similar to the EU4Health programme that aims the ‘exchange of best practices, supporting networks for knowledge sharing or mutual learning (.) unlocking the potential of innovation in health’, while the Expert Panel on Effective Ways of Investing in Health recommends to invest in ‘learning communities within and across Member States to share lessons learned’. Primary care is well-networked with the Global Organisation of Family Doctors (WONCA), the European General Practice Research Network (EGPRN) and the European Form for Primary Care (EFPC). These networks may play a role in a concerted reaction to the calls on health systems and services. It is up to the international primary care community to take this up.
